# Laxative effects of partially defatted flaxseed meal on normal and experimental constipated mice

**DOI:** 10.1186/1472-6882-12-14

**Published:** 2012-03-09

**Authors:** Jiqu Xu, Xiaoqi Zhou, Chang Chen, Qianchun Deng, Qingde Huang, Jin'e Yang, Nianhong Yang, Fenghong Huang

**Affiliations:** 1 Department of Product Processing and Nutriology, Oil Crops Research Institute, Chinese Academy of Agricultural Sciences, 2 Xudong Second Road, Wuhan 430062, People's Republic of China; 2 Hubei Key Laboratory of Lipid Chemistry and Nutrition, Oil Crops Research Institute, Chinese Academy of Agricultural Sciences, 2 Xudong Second Road, Wuhan 430062, People's Republic of China; 3 Department of Nutrition and Food Hygiene, School of Public Health, Tongji Medical College, Huazhong University of Science and Technology, 13 Hangkong Road, Wuhan 430030, People's Republic of China; 4 Department of Gastroenterology, The No.1 Hospital of Yichang, 2 Jiefang Road, yichang 443000, People's Republic of China

**Keywords:** Flaxseed, Constipation, Diphenoxylate, Dietary fibers, Laxative

## Abstract

**Background:**

Constipation is a very common health problem in the world. Intake of sufficient amount of dietary fibers is a cornerstone in the prevention and treatment of constipation. As a traditional medicine, flaxseed has been used to treat constipation for centuries, but the controlled trials are rare. The purpose of the present study was to assess that whether partially defatted flaxseed meal (PDFM) has the potential role to facilitate fecal output in normal and experimental constipated mice.

**Methods:**

After supplemented with 2.5%, 5% and 10% (w/w) PDFM (L-, M- and H -PDFM) for 14 days, the constipation models of mice were induced by atropine-diphenoxylate. The small intestinal transit rates, start time of defecation, amount of defecation and wet weight of feces were researched in normal and constipation model mice.

**Results:**

M- and H-PDFM significantly increase small intestinal transit rates in constipation model mice. All dose of PDFM markedly shortened the start time of defecation and M- and H-PDFM significantly increase stool frequency and weight in both normal and constipation model mice.

**Conclusions:**

PDFM may be a useful laxative to facilitate fecal output in normal and constipation conditions.

## Background

Constipation is a worldwide public health problem in both developed and developing countries. In general, constipation is defined as infrequent or difficult passage of stool. It significantly affects the quality of life because constipation can cause not only discomfort and restlessness but also abdominal distension, vomiting, restlessness, gut obstruction, and perforation and even is associated with fatal pulmonary embolism [1]. In North America, the most estimates of the prevalence of constipation range from 12% to 19% [2]. In addition, constipation increases with increasing age [2]. A lot of drugs have been approved for the treatment of constipation and most of them are laxatives. Unfortunately, these drugs are not so ideal in clinical use because of their potentially adverse side effects such as inducing tolerance, melanosis coli, or cathartic colon [3]. Therefore, increasing fibers ingestion including dietary or medicinal fibers has been well accepted to be a primary treatment modality to relieve symptoms, especial for mild complaints of infrequency or hard stools [3].

Flax is one of the most important oilseed crops of the world and flaxseed is a main source of α-linolenic acid (18:3; n-3) and the richest food source of lignans. The health benefits of both substances have been well-documented [4-6]. Besides, flaxseed is also a good source of soluble and insoluble fibers and has been used as a traditional medicine for centuries to treat constipation. Flaxseed exists in several main forms such as whole seed, ground seed and partially defatted flaxseed meal (PDFM), and PDFM contains the highest content of dietary fibers in common forms of flaxseed [7]. Some investigations had shown that flaxseed exhibited the similar laxative actions in healthy [8,9] and constipated subjects [10], whereas the relevant controlled trials are rare. In the present study, we tried to assess that whether PDFM has the potential role to facilitate fecal output in normal and experimental constipated mice.

## Methods

### Animals and diets

Male Kunming mice (20-24 g, provided by the Experimental Animal Center of Tongji Medical College, Huazhong Science and Technology University.) were used for this experiment. The animals were housed 5 per cage and maintained at a controlled ambient temperature (24 ± 1°C) under diurnal conditions (light-dark: 08:00-20:00). All mice were allowed access to standard diet (AIN-93 M) and tap water *ad libitum*. The animals were cared for in accordance with *the Guiding Principles in the Care and Use of Animals*. The experiment was approved by the Oil Crops Research Institute Council on Animal Care Committee, Chinese Academy of Agricultural Sciences. Flaxseed was partly defatted by cold-pressing and ground and then stored in nitrogen. The PDFM contained approximately 10% of flaxseed oil.

### Measurement of PDFM on small intestinal transit rates in normal mice

The rationale and method for measuring small intestinal transit rates are described elsewhere [11,12]. Briefly, forty animals were randomly divided into four groups (n = 10 each): control group (CON), low, middle and high dose PDFM groups (L-, M- and H-PDFM). Mice in control group were fed the standard diet and the L-, M- and H-PDFM groups received standard diet supplemented with 2.5%, 5% and 10% (w/w) PDFM for 14 days, respectively. After fasting for 16 h with free access to water, all mice were placed in small transparent cages individually and allowed access to their diets. Five minutes later these animals were administered with distilled water (0.1 ml/10 g) suspension containing 5% charcoal and 10% gum acacia through intragastric gavage. The mice were killed by exarticulation at 25 min after the charcoal meal administration. The small intestine from the pylorus to the caecum was quickly removed and the distance traveled by the charcoal meal and the total length of the intestine were measured. The small intestinal transit rate was evaluated by charcoal powder propelling ratio which was calculated as the percentage of the distance traveled by the charcoal meal relative to the total length of the small intestine [11].

### Measurement of PDFM on fecal output character in normal mice

Administering of various doses of PDFM and the four experimental groups were in accordance with Section 2.2. After fasting for 16 h with free access to water, all mice were administered with distilled water (0.1 ml/10 g) suspension containing 5% charcoal and 10% gum acacia through intragastric gavage. Then the animals were immediately placed in small transparent cages individually and allowed access to their diets and tap water *ad libitum*. The length of time from charcoal meal administration to the appearance of first darkened defecation was recorded. Feces were collected, counted and weighed for 8 h after intragastric gavage administration.

### Measurement of PDFM on small intestinal transit rates in diphenoxylate-induced constipated mice

Fifty animals were randomly divided into five groups (n = 10 each): control group (CON), atropine-diphenoxylate group (AD) and L-, M- and H-PDFM groups. Mice in control and AD groups were fed the standard diet and the L-, M- and H-PDFM groups received standard diet supplemented with 2.5%, 5% and 10% (w/w) PDFM for 14 days, respectively. After fasting for 16 h with free access to water, mice in control group were administered with normal saline and the other animals were treated with atropine-diphenoxylate (diphenoxylate 5 mg/kg BW; atropine sulfate 0.05 mg/kg BW). Then 25 min after the atropine-diphenoxylate was administered, all animals were placed in small transparent cages individually and all experimental processes were performed in accordance with Section 2.2.

### Measurement of PDFM on fecal output character in diphenoxylate-induced constipated mice

Administering of various doses of PDFM and the five experimental groups were in accordance with Section 2.4. After fasting for 16 h with free access to water, all experimental processes were performed in accordance with Section 2.3.

### Statistical analysis

Values are presented as mean ± SEM (standard error of the mean). The measurement data were analyzed by one-way ANOVA, followed by the Fisher PLSD post hoc test if the overall differences were significant (*P *< 0.05). All statistical analyses were performed using SPSS 13.0 statistical software (SPSS Inc., Chicago, IL) and a difference was considered significant when *P *< 0.05.

## Results

### Effect of PDFM on small intestinal transit rate in normal mice

The effect of PDFM on the small intestinal transit rate was evaluated by charcoal powder propelling ratio. Although all three PDFM groups appeared to reveal a higher charcoal powder propelling ratio than control group, the differences are not significant (data not shown).

### Effect of PDFM on fecal output character in normal mice

**Figure 1:**
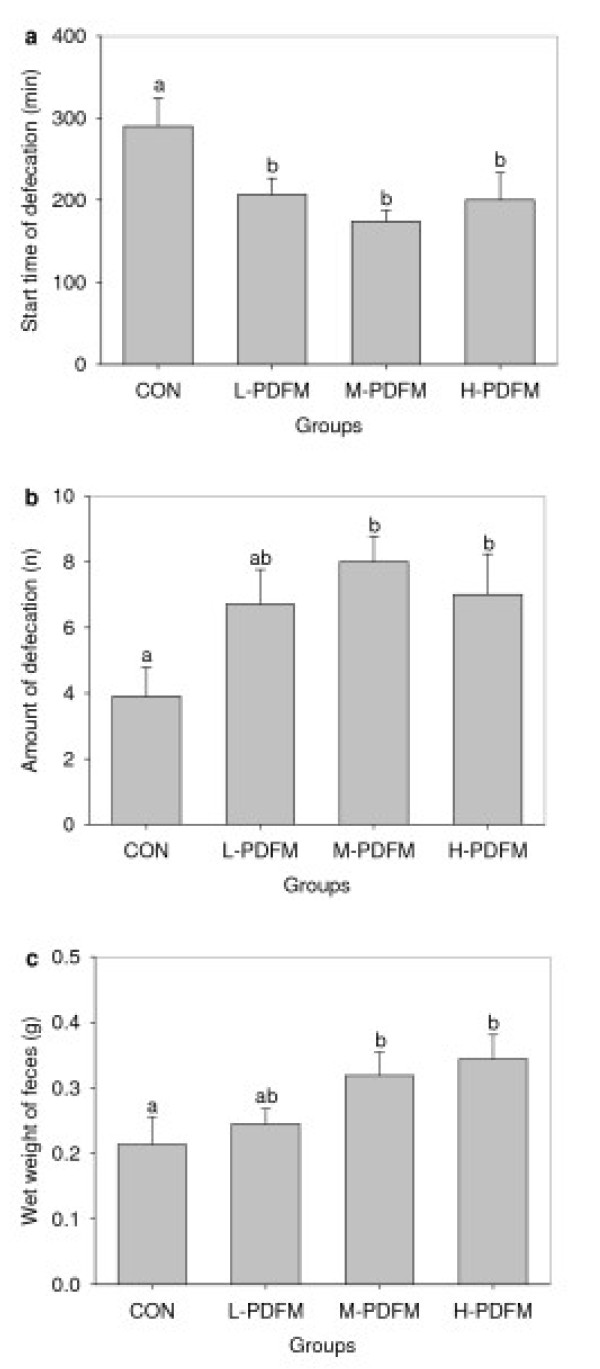
**Effect of PDFM on fecal output character in normal mice**. CON: the control group; L-, M- and H- PDFM: L-, M- and H- PDFM groups. Bars represent the mean ± SEM. n = 10. Groups sharing different letters above the bars mean statistically significant differences (P < 0.05), while those denoted by any same letters are insignificantly.

The effect of PDFM on fecal output character in normal mice is shown in Figure 1a, b and 1c. The time of first fecal output with a charcoal meal in all three doses of PDFM groups were significantly shorter than that in control group. Mice treated with M- and H- PDFM exhibited a significantly increased amount of defecation and wet weight of feces when compared with the control animals.

### Effect of PDFM on small intestinal transit rate in diphenoxylate-induced constipated mice

**Figure 2:**
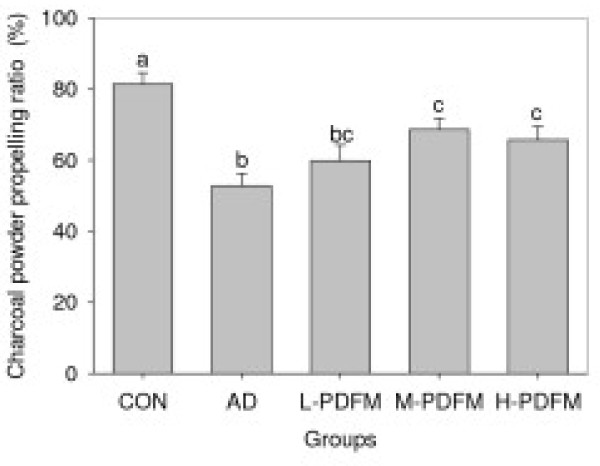
**Effect of PDFM on small intestinal transit rates in diphenoxylate-induced constipated mice**. CON: the control group; AD: atropine-diphenoxylate group; L-, M- and H-PDFM: L-, M- and H- PDFM groups. Bars represent the mean ± SEM. Bars represent the mean ± SEM. n = 10. Groups sharing different letters above the bars mean statistically significant differences (P < 0.05), while those denoted by any same letters are insignificantly.

As can be seen from Figure 2, AD group showed markedly lower charcoal powder propelling ratio in comparison with the control group. Although the charcoal powder propelling ratios in all three PDFM groups were also significantly less than that in control group, the M- and H-PDFM groups had significantly higher these ratios than did AD group.

### Effect of PDFM on fecal output in diphenoxylate-induced constipated mice

**Figure 3:**
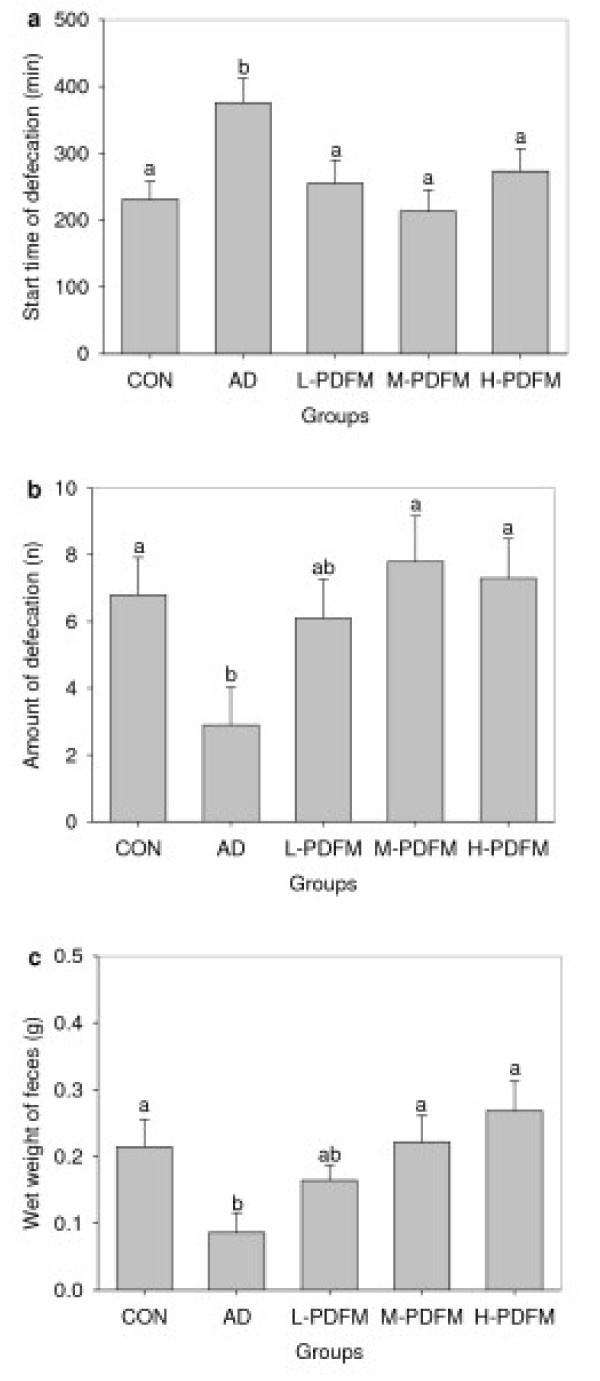
**Effect of PDFM on fecal output in diphenoxylate-induced constipated mice**. CON: the control group; AD: atropine-diphenoxylate group; L-, M- and H- PDFM: L-, M- and H-PDFM groups. Bars represent the mean ± SEM. Bars represent the mean ± SEM. n = 10. Groups sharing different letters above the bars mean statistically significant differences (P < 0.05), while those denoted by any same letters are insignificantly.

As can be seen from Figure 3a, b and 3c, the time of first fecal output with a charcoal meal in AD group were significantly longer than that in control group. Treatment with PDFM reversed the increase in the time when compared with AD and reached the level of control group. In addition, mice in AD group also showed the marked decline in amount of defecation and wet weight of feces than did control subjects, whereas animals in M- and H-PDFM groups revealed significantly more amount of defecation and wet weight of feces than that in AD group and were similar to their young counterparts.

## Discussion

Constipation is a very common health problem which significantly affects the quality of life among those who suffer from this condition. It is well known that fibers have an important role in occurrence and development of this disease, whereas the total dietary fibers intake in adults appears to be much less than the recommended level. Therefore, although some drugs and other modalities (e.g. biofeedback, surgery) have been used to treat constipation, intake of sufficient amount of dietary fibers is still a cornerstone in the prevention and treatment of this disease [13].

Traditionally, dietary fibers were defined as the portions of plant foods that were resistant to digestion by human digestive enzyme [14]. In general, dietary fibers are divided into two basic types: insoluble fibers and soluble fibers. Soluble fibers such as pectin and guar gum dissolve in water and usually form a gel while insoluble fibers such as cellulose, hemicellulose and lignin do not dissolve in water. Dietary fibers intake provides numerous health benefits, including cardiovascular health promotion, diabetes prevention, obesity prevention and gastrointestinal function improvement [14].

As a functional food source [13,15,16], flaxseed has been the focus of considerable interest in the fields of diet and disease research. In addition to being one of the richest of α-linolenic acid and lignan, flaxseed is also an important source of dietary fibers (35-45%) of which the proportion of soluble to insoluble fibers in flaxseed varies between 1:4 and 2:3 [13,15]. Although there is no consensus about the effect of soluble fibers on gastrointestinal transit [13,17,18], insoluble fibers which inhibit intestinal digestive processes result in decreased transit times within stomach and small intestine [19]. Moreover, the swelling property of insoluble fibers can cause chyme to have a larger bulk in the intestines. In fact, the laxative actions of dietary fibers from natural food sources, which are mixtures of soluble to insoluble fibers, are entirely dependent on luminal bulk [19]. In the present study, despite the fact that PDFM only had a trend to promote small intestinal transit in normal mice, it significantly shortened the small intestinal transit time for diphenoxylate-induced constipated animals. These meant that PDFM had a role in increasing small intestinal transit rates. It should be noted that the majority of time for whole gut transit occurs within the large intestine. When the contents move into the large intestine, insoluble fibers remains largely unfermented and retains water by acting as "sponges" which increase the bulk modulus and soften stools, whereas most of soluble fibers and a limited extent of insoluble fibers were fermented by colonic microflora which provides a readily usable substrate for the stimulation of microbial growth and as a result, increases the bacterial mass in the colon. It has been well documented that bacteria also represent a much larger proportion of the fecal mass [20,21]. The fermentation process of dietary fibers also forms short-chain fatty acids such as acetate, propionate, and butyrate [22]. These short-chain fatty acids are the major anions in the large intestine and regulate various colonic functions [23]. There is a large and growing body of evidence showing that physiological concentration of these short-chain fatty acids significantly increases the colonic motility and stimulates colonic transit through various mechanisms [24-26]. Although many factors can affect the producing of short-chain fatty acids, which makes the levels of short chain fatty acids were unstable, more consumption of soluble fibers leads to higher short-chain fatty acids concentration [27,28]. In addition, additional laxative effect may also attribute to the residue oil that is left in the flaxseed meal in this study by its lubricant property [13]. As results of these regulation mechanisms, in the present study, PDFM supplementation markedly decreased gastrointestinal transit time as well as increased stool frequency and weight in both normal and diphenoxylate-induced constipation conditions.

## Conclusions

In conclusion, the present results indicate that PDFM has the ability to promote intestinal motility, stimulate intestinal transit as well as increase stool frequency and weight. These results suggest that PDFM may be a useful laxative to facilitate fecal output in normal and constipation conditions.

## Competing interests

The authors declare that they have no competing interests.

## Authors' contributions

JX designed and wrote a first draft of the paper. XZ, QD and JY carried out all the experiments. CC participated in the design of the study. QH performed the data analysis and created the figures. FH and NY contributed to the design of the study, reviewed the manuscript and contributed to the final version. All authors contributed to and have approved the final manuscript.

## Pre-publication history

The pre-publication history for this paper can be accessed here:

http://www.biomedcentral.com/1472-6882/12/14/prepub
